# Relative contributions of auditory and cognitive functions on speech recognition in quiet and in noise among older adults^☆^

**DOI:** 10.1016/j.bjorl.2018.10.010

**Published:** 2018-12-10

**Authors:** Siti Zamratol Mai Sarah Mukari, Yusmeera Yusof, Wan Syafira Ishak, Nashrah Maamor, Kalaivani Chellapan, Mariam Adawiah Dzulkifli

**Affiliations:** a Universiti Kebangsaan Malaysia, Institute of Ear, Hearing and Speech, Kuala Lumpur, Malaysia; b Universiti Kebangsaan Malaysia, Faculty of Heath Sciences, Kuala Lumpur, Malaysia; c Ministry of Health, Putrajaya, Malaysia; d Universiti Kebangsaan Malaysia, Faculty of Engineering & Built Environment, Bangi, Malaysia; e International Islamic University, Kuliyyah of Islamic Revealed Knowledge and Human Sciences, Kuala Lumpur, Malaysia

**Keywords:** Speech recognition, Hearing threshold, Auditory, Cognition, Elderly, Reconhecimento de fala, Limiar auditivo, Auditivo, Cognição, Idoso

## Abstract

**Introduction:**

Hearing acuity, central auditory processing and cognition contribute to the speech recognition difficulty experienced by older adults. Therefore, quantifying the contribution of these factors on speech recognition problem is important in order to formulate a holistic and effective rehabilitation.

**Objective:**

To examine the relative contributions of auditory functioning and cognition status to speech recognition in quiet and in noise.

**Methods:**

We measured speech recognition in quiet and in composite noise using the Malay Hearing in noise test on 72 native Malay speakers (60–82 years) older adults with normal to mild hearing loss. Auditory function included pure tone audiogram, gaps-in-noise, and dichotic digit tests. Cognitive function was assessed using the Malay Montreal cognitive assessment.

**Results:**

Linear regression analyses using backward elimination technique revealed that had the better ear four frequency average (0.5–4 kHz) (4FA), high frequency average and Malay Montreal cognitive assessment attributed to speech perception in quiet (total *r*^2^ = 0.499). On the other hand, high frequency average, Malay Montreal cognitive assessment and dichotic digit tests contributed significantly to speech recognition in noise (total *r*^2^ = 0.307). Whereas the better ear high frequency average primarily measured the speech recognition in quiet, the speech recognition in noise was mainly measured by cognitive function.

**Conclusions:**

These findings highlight the fact that besides hearing sensitivity, cognition plays an important role in speech recognition ability among older adults, especially in noisy environments. Therefore, in addition to hearing aids, rehabilitation, which trains cognition, may have a role in improving speech recognition in noise ability of older adults.

## Resumo

**Introdução:**

A alteração da acuidade auditiva, do processamento auditivo central e da cognição são fatores que contribuem para a dificuldade de reconhecimento da fala em idosos. Portanto, quantificar a contribuição desses fatores no problema de reconhecimento da fala é importante para a formulação de uma reabilitação holística e efetiva.

**Objetivo:**

Examinar as contribuições relativas do funcionamento auditivo e do estado cognitivo para o reconhecimento da fala no silêncio e no ruído.

**Método:**

Nós medimos o reconhecimento de fala no silêncio e no ruído composto com o teste *Malay hearing in noise test* em 72 idosos malaios nativos falantes (60-82 anos) com audição normal a perda auditiva de grau leve. A avaliação da função auditiva incluiu audiograma de tons puros, teste *gaps-in-noise* e testes dicótico de dígitos. A função cognitiva foi avaliada pelo teste *Malay Montreal cognitive assessment*.

**Resultados:**

Análises de regressão linear com técnicas de eliminação *backward* na orelha melhor revelaram média de quatro frequências (0,5–4 kHz) (4AF), média de alta frequência e teste *Malay Montreal cognitive assessment* na orelha melhor, mensurada pela percepção da fala no silêncio (r^2^ total = 0,499). Por outro lado, a média de alta frequência, *Malay Montreal cognitive assessment* e o teste dicótico de dígitos contribuíram significativamente para o reconhecimento da fala no ruído (r^2^ total = 0,307). Enquanto a média de alta frequência da melhor orelha mediu principalmente o reconhecimento da fala no silêncio, o reconhecimento da fala no ruído foi mensurado principalmente pela função cognitiva.

**Conclusões:**

Esses achados destacam o fato de que, além da sensibilidade auditiva, a cognição desempenha um papel importante na capacidade de reconhecimento da fala em idosos, principalmente em ambientes ruidosos. Portanto, além de aparelhos auditivos, a reabilitação, que treina a cognição, pode ter um papel na melhoria da capacidade do reconhecimento da fala no ruído entre os idosos.

## Introduction

Following a speech conversation in a noisy environment is hard, especially among older adults with and without hearing impairment.^1^ Three hypotheses have been proposed to explain the speech understanding difficulties in older adults; they are peripheral auditory, central auditory and cognitive processes.^2^ Although the contributions of central auditory and cognitive processes on speech recognition performance in adverse listening conditions have been documented,^3^, ^4^ management of hearing impairment has been mostly focused towards improving audibility through the use of hearing amplification devices. A more holistic rehabilitation approach addressing the processes involved in speech recognition difficulties is necessary in order to provide a more satisfactory result.

In addition to peripheral hearing loss, ageing is often accompanied by reduced sound processing efficiency due to degeneration of central auditory pathway^5^ and a decline in cognitive function.^6^ Of the three factors, peripheral hearing loss has been extensively studied as the underlying factor for speech-in-noise difficulties experienced by older adults.^7^ Although those studies suggest that peripheral hearing loss plays a primary role in speech understanding difficulties in noise, the fact that older listeners, despite normal hearing, tend to have greater difficulties than young ones,^8^, ^9^ indicates that processes other than peripheral hearing also contribute to these speech recognition difficulties.

The roles of central auditory and cognitive factors in understanding speech in noise have been extensively studied in the past decades.^10^, ^11^ Most of those studies have included measures of auditory temporal resolution.^7^, ^8^, ^11^ The effects of temporal processing deficits on speech recognition remain unclear. For example, Besser et al.^11^ who examined the contributions of auditory, cognitive and linguistic abilities to the listening in spatialized noise-sentences (LISN-S) outcomes in normal hearing young and older groups found that measures of temporal processing, linguistic abilities and hearing level did not predict LISN-S outcomes in the older age group. In contrast are the results of Tyler et al.^12^ who reported significant correlation between gap thresholds and word recognition in noise after combining the data of the young and older adults. It should be noted, however, that across studies, differences in the ranges of age and hearing level of the study participants, measures of temporal processing as well as variances in the speech recognition materials may have contributed to the difference in results.

Another central auditory processing function which declines with age is dichotic listening. Dichotic listening is a challenging task in that a listener is required to cope with competing sound signals presented to both ears simultaneously. A study investigated the influence of age attentional control of bottom-up, stimulus driven lateral effect in younger and older adults.^13^ Study subjects were tested using free recall, forced right and forced left listening paradigms. Their results revealed that both young and older groups showed better performance in the right ear (right ear advantage) in free recall and forced right listening paradigms. However, in forced left listening paradigm, while the younger group demonstrated left ear advantage, this phenomenon was not observed in older group, suggesting that ageing reduces the ability for top-down attentional control of a bottom up laterality effect. Since attention is known to reduce with age^14^ and is expected to be generally important for speech in noise performance,^15^ it is possible that dichotic listening influences speech recognition performance in noise. Additionally, studies on dichotic listening using speech stimuli have shown that ageing is associated with an overall reduction on both right and left ear scores, with the left ear score showing more rapid reduction resulting in a greater right ear advantage.^16^ It is suggested that right ear advantage in linguistically based dichotic listening may have considerable impact in binaural signal processing, which may interfere with the ability recognizing speech in noise.^17^ Despite the possible influence of dichotic listening on speech recognition in noise, a review of the relevant literature reveals very limited studies which included a dichotic test.

The influence of cognition on speech understanding in noise has been vastly studied and established.^10^, ^18^ Among the commonly studied cognitive measures included working memory capacity, attention and speed of processing.^19^, ^20^, ^21^ A meta-analysis, which involved 25 articles that included attention, memory, executive function, intelligence quotient and processing speed revealed a general association between cognition and speech-in-noise performance.^22^ While most studies examining the influence of cognition on speech performance in noise used domain-specific cognitive assessment, a few studies such as Besser et al.^11^ and Zhan et al.^23^ have used a screening test for global cognitive status such as the Montreal cognitive assessment (MoCA). Their studies demonstrated a significant influence of MoCA on speech performance in noise.

Given that difficulties listening in noise is common and continue to decline among older adults, and the fact that hearing loss alone does not always explain these problems, it is important that we examine the relative contributions of peripheral auditory, central auditory and cognitive processes to speech recognition. Knowing the magnitude of the effects of these processes is crucial in developing a more comprehensive management plan of hearing loss in older adults. Therefore, this study is aimed to determine the relative contribution of auditory and cognitive functions on speech recognition in older adults with normal to mild hearing loss. This study is novel in a way. Firstly, while many studies have examined the contribution of central auditory processing on speech recognition performance among older adults, very few have included dichotic listening test. We included gaps-in-noise and dichotic listening test as measures of central auditory processing in this study. Specifically, we view the inclusion of dichotic listening test as important because some studies suggest the potential of dichotic listening training in improving speech recognition.^24^ We hypothesized that speech recognition relies on hearing level, central auditory processing and cognitive functions, and that each of the factors contribute differently in varied HINT conditions.

## Methods

### Participants

A total of 72 participants participated in this study. To be included participants must be 60 years old or more, right handed, native Malay speakers had no history of stroke, head injury, ear surgery, psychiatric problem or dementia. Additionally, participants must have normal middle ear function based on tympanometry and symmetrical hearing level with normal hearing or mild hearing loss as indicated by Pure Tone Average (PTA) for 0.5, 1, 2 and 4 kHz of 40 dB HL or less in the better ear. Symmetrical hearing threshold was defined as difference between the PTA (0.5–4.0 kHz) of both ears of no greater than 10 dB. The tests were spread into two sessions, which lasted about one hour each. The experimental protocol and procedures in this study were approved by the Universiti Kebangsaan Malaysia Research Ethics Committee (approval n°. NN-050-2015). Informed consent was obtained before data collection.

### Peripheral hearing tests

#### Pure tone audiogram

Pure-Tone Audiometry (PTA) was conducted by a trained audiologist in a sound treated booth, using standard TDH-39 earphones and a Madsen OB822 audiometer (Madsen Electronics Itera 2). Air conduction (AC) thresholds for each ear were measured for frequencies of 0.5, 1.0, 2.0, 4.0, and 8.0 kHz. Bone Conduction (BC) was evaluated whenever AC thresholds were greater than 20 dB HL for frequencies of 0.5, 1.0, 2.0 and 4.0 Hz. Masking for air and bone conduction thresholds were performed when indicated. The four-frequency pure tone average (4FA) was calculated for frequencies of 0.5, 1.0, 2.0 and 4.0 kHz. High frequency average (HFA) was based on frequencies 4 and 8 kHz. Normal and mild hearing loss were defined as follows: no impairment, PTA ≤25 dB HL and mild, PTA 26–40 dB HL.^25^ The better ear (BE) 4FA and BE HFA were used in the analyses.

#### Tympanometry

Tympanometry was conducted on both ears using an Interacoustic Titan tympanometer. A normal tympanogram was defined as static admittance of 0.2–1.5 mmho and tympanometric width of 35–125 dapa.^26^

### Central auditory tests

#### Dichotic digits test (DDT)

DDT was conducted using the Malay double dichotic digits test.^27^ Pairs of different digits were presented simultaneously to each of the two ears at 35 dB sensation level (dB SL). The DDT was conducted using free recall test condition, in which the subject was instructed to repeat all the four digits that they heard in no specific order. The test was carried out using 20 stimulus items; each item consists of four different digits. A correct response was allocated to each digit that was repeated correctly. The possible total correct score was 40 for each ear. The Right Ear Score (RES) and the Left Ear Score (LES) were calculated as percent correct. The Right Ear Advantage (REA), determined by deducting the left ear score from the right ear score was used in the analysis.

#### Gaps-in-noise test (GIN)

The GIN test was administered and scored according to the set criteria by Musiek et al.^28^ The GIN contains a series of 36 different 6-s white noise segments. Each of the white noise segments contain between 0 and 3 silence gaps of silence ranging from 2 to 20 ms. The GIN contains 60 gaps and the order of the gap durations is randomized. A 5-s gap of silence separates each 6-s noise segment. The test was conducted binaurally through a headphone at 35 dB SL. The subject was asked to listen and respond to the silence gap that occur within each noise burst. GIN threshold was defined by the shortest silence gap that was correctly detected by the subject at least four out of six times.

### Hearing in noise test (HINT)

The speech in noise test was conducted in a sound booth using the Malay version of Hearing in Noise Test (MyHINT).^29^ MyHINT is an adaptive open-set speech recognition test, which is used to measure the Reception Threshold for Sentences (RTSs). HINT is equipped with an audio sound processing that simulates the source locations for the speech at 0° and noise at either 0°, 90°, and 270° azimuths.^30^ The My HINT consists of 12 phonemically balanced lists of 20 short 4–6 words simple sentences. The noise used in HINT is steady-state speech-shaped noise.

The HINT was conducted in four different conditions: (1) In quiet (HINT Q), (2) HINT Noise Front (HINT NF), (3) HINT Noise Right (HINT NR), and HINT Noise Left (HINT NL). In all test conditions, speech was presented from the front. In noise conditions, the noise level was fixed at 65 dB A. RTS was calculated based on the average presentation level of the 5th through the 20th sentences and the level at which the 21st sentence would be presented.

In this study, HINT (Q) and HINT (composite), a score often used as a descriptor of noise conditions^31^ were analyzed. HINT (composite) was calculated based on the three RTS measures: RTS for HINT (Composite) = [2 HINT (NF) + HINT (NR) + HINT (NL)]/4.

### Cognitive assessment

The global cognitive function was assessed using the Malay version of Montreal cognitive assessment,^32^ which was adapted from the original MoCA by Nasreddine et al.^33^ It consists of 16 items, which covers attention and concentration, executive functions, memory, language, visuo-constructional skills, conceptual thinking, calculations, and orientation. MoCA has the possible full score of 30. In this study, raw score was used. The MoCA is only a screening test for cognition and does not provide an in depth measure of cognition.

### Statistical analysis

Data was analyzed using the Statistical package for the social sciences version 22. Descriptive analyses were performed on demographic data, auditory function tests and MoCA. The associations between measures on HINT test with age, peripheral auditory, central auditory tests and MoCA were assessed using Pearson Correlation test. Finally, multiple linear regression analyses were used to determine relative contributions of auditory performance, and global cognitive performance and HINT measures.

## Results

**Figure 1:**
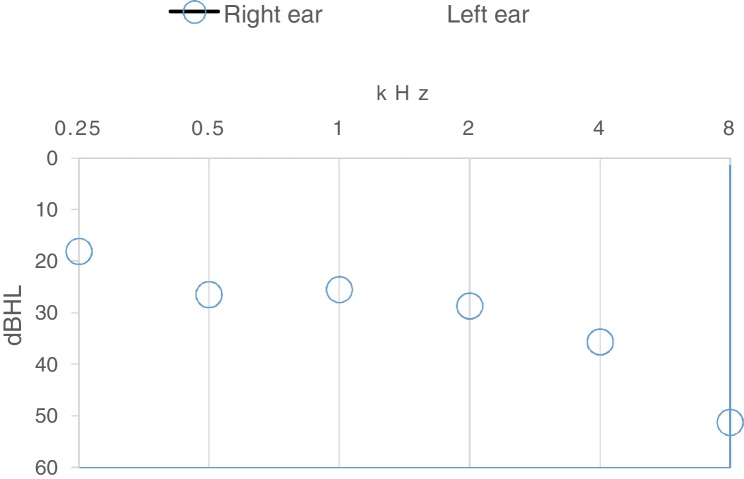
The mean hearing thresholds for the right and left ears.

A total of 72 individuals, 18 males and 54 females between 60 and 82 years old (Min. 67.4; SD = 4.9 years) participated. Table 1 shows the means and standard deviations of MoCA, 4FAs and HFAs of the participants. Fifty percent of the participants had normal hearing (4FA ≤ 25 dB HL) and another 50% had mild hearing loss. The BE HFA ranged from 10 to 80 dB HL; less than 25 dB HL (18.1%), between 26 and 40 dB HL (30.6%), between 41 and 70 dB HL (48.6%) and more than 70 dB HL in (2.8%) of the participants. Fig. 1 shows the mean hearing thresholds across the test frequencies for both ears. The audiogram indicates a fairly flat configuration up to 2.0 kHz and gently sloping at higher frequencies, typical of presbycusis audiogram.

**Table 1:** Means and standard deviations of MoCA, 4FA and HFA (*n* = 72).

Variables	Mean (SD)
MoCA	17.7 (4.5)
RE 4FA (dB HL)	26.5 (8.0)
LE 4FA (dB HL)	26.6 (7.6)
BE 4FA (dB HL)	24.9 (7.5)
RE HFA (dB HL)	44.1 (16.9)
LE HFA (dB HL)	46.1 (16.9)
BE HFA (dB HL)	41.1 (15.5)

### Hearing in noise test and central auditory tests

Table 2 summarizes the means and standard deviations of HINT (Q), HINT (composite), DDT (REA) and GIN. The intercorrelations between HINT tests and the predictor variables were shown in Table 3. The results indicated that higher BE 4FA, BE HFA and lower MoCA were associated with worse HINT (Q) and HINT (composite). In addition, HINT (composite) was also positively correlated to GIN. There was a positive correlation between HINT (Q) and HINT (composite).

**Table 2:** Means and standard deviations of HINT (Q), HINT (composite), dichotic digit test and gaps-in-noise.

Variables	Mean (SD)
HINT (Q) (dB A)	28.7 (5.9)
HINT (C) (dB SNR)	−5.5 (2.1)
DDT (REA) (%)	16.7 (13.5)
Gaps-in-noise (ms)	13.7 (4.2)

**Table 3:** Correlation analysis between reception thresholds of sentences of HINTs, age, MoCA, peripheral and central auditory tests.

	HINT (Q)	HINT (C)	BE 4FA	BE HFA	GIN	DDT (REA)	MoCA
HINT (Q)	–	0.505^a^	0.530^a^	0.621^a^	0.138^c^	0.137^c^	−0.326^b^
HINT (Composite)		–	0.235^b^	0.368^a^	0.234^b^	0.199^c^	−0.436^a^
BE 4FA			–	0.413^a^	0.048^c^	0.061^c^	−0.262^b^
BE HFA					0.205^c^	−0.067^c^	−0.182^c^
GIN					–	0.166^c^	−0.206^c^
DDT (REA)						–	0.065^c^
MoCA							–

^a^*p* < 0.001; ^b^*p* < 0.05; ^c^*p* > 0.05.

### Contributions of auditory functions and cognitive function to hearing in noise test

We performed multiple linear regression analyses to determine how much hearing acuity, central auditory tests and MoCA attributed to the variations of each of the HINT outcomes. To examine potential cases of collinearity between predictor variables, we performed a diagnostic collinearity test using a linear regression analysis. No collinearities were observed, with variance inflation factor values less than 2. Thus, all predictor variables were included in the regression analyses.

Multiple linear regression analyses were performed using backward elimination method. The predictor variables were excluded stepwise, removing the variable with the highest *p* value at each step, until only variables with *p* < 0.1 remained in the model. The final models are shown in Table 4.

**Table 4:** Multiple regression models for outcome measures. The last column indicates the *R*^2^ of the regression models and relative contribution (part *r*^2^) of each independent variable to the variances of dependent variables.

	*B* coefficient	*t*	*p*-Value	Part *R*^2^	Total *R*^2^
HINT (Q)					0.499
BE 4FA	0.272	2.870	0.005	0.109	
BE HFA	0.488	5.246	0.000	0.292	
MoCA	−0.177	−2.017	0.048	0.057	
HINT (Composite)					0.307
BE HFA	0.312	3.097	0.003	0.124	
DDT	0.245	2.473	0.016	0.082	
MoCA	−0.396	−3.393	0.000	0.185	

The results indicated that HINT (Q) was best predicted by BE 4FA, BE HFA and MoCA, *F*(4.67) = 18.706, *p* < 0.001. The three variables accounted to 49.9% of the variance. The proportion of variance in RTS (Quiet) uniquely explained by these variables, as indexed by the squared semi-partial correlations (part *r*^2^) were 29.2% by the BE HFA, 10.9% by the BE 4FA, and 5.7% by MoCA. The positive beta coefficient for the two hearing averages and negative beta coefficient for MoCA indicate that HINT (Q) performance worsened as the hearing averages increased and the MoCA reduced. Individual differences in BE HFA, DDT (REA), and MoCA together accounted for 30.7% of the HINT (Composite) variance, *F*(3.68) = 11.467, *p* < 0.001. MoCA uniquely accounted to 18.5% of the variance, followed by BE HFA (12.4%) and DDT (REA) (8.2%). Lower MoCA, greater BE HFA and DDT (REA) yielded worse HINT (COMPOSITE) performance.

## Discussion

### Contributions of audiometric thresholds to HINT outcomes

In the present study, which involved older adult participants with normal and with mild hearing loss at 4FA, hearing acuity at both low to mid frequencies as well as at high frequencies significantly contributed to the variance of HINT in quiet. The 4FA and HFA uniquely accounted for 10.9% and 29.2% of the variance of HINT (Q), respectively. These results corroborated the findings of previous research which explained the importance of speech spectrum audibility in speech recognition performance.^7^, ^34^

The importance of high frequency hearing acuity in speech recognition in quiet, especially in older adults, has been well established. Articulation index theory predicts that individuals with sloping high frequency hearing loss such as in presbycusis will experience difficulty listening to speech at conversational level due to inaudibility of high frequency speech sounds.^35^ Apart from being important for detecting weak fricatives such as /f/ and /s/ sounds, high frequency hearing acuity is also essential in differentiating voiced versus voiceless cues and place-of-articulation cues.^36^ Furthermore, it is well known that hearing loss in a given frequency region affects not only audibility but may also affects suprathreshold auditory processing abilities such as intensity, frequency and temporal processing in the frequency region corresponding to hearing loss.^37^ Suprathreshold processing dysfunction may spread to frequencies lower than that affected by hearing loss as well causing an off-channel impact on intensity, frequency and temporal processing.^38^, ^39^, ^40^

The importance of high frequency hearing in speech intelligibility in noise has also been well documented. For example, a recent study indicated that elderly listeners with better high frequency hearing in the region between 6 and 10 kHz had greater spatial release from masking resulting in better speech recognition when speech and noise are spatially separated.^11^ Another study has shown that optimal speech recognition in noise is obtained by extending audibility up to 7 kHz.^41^ The finding of the present study which indicate a significant relationship between BE HFA and HINT (Composite) are in line with those studies.

Although most prior studies have observed strong correlations between speech recognition in both quiet and noise and hearing thresholds up to 4 kHz,^42^, ^43^ it is not the case in the present study. This observation could partly be due to the fact that in the present study, the participants’ BE 4FA was relatively more homogenous ranging from 3 to 40 dB HL compared to that of BE HFA which were remarkably more varied, ranging from 5 to 90 dB HL. Furthermore, it is also possible that averaging hearing thresholds of 2 and 4 kHz with the largely age-independent thresholds of 0.5 and 1 kHz weakened the relationship between the 4FA and speech recognition measure, especially in view of the sloping audiogram configuration seen in this study.

### Contributions of central auditory processing to HINT outcomes

GIN and DDT, were included as measures of central auditory processing in the present study. Generally, our findings revealed that GIN did not contribute to any of the HINT outcomes. In contrast, the DDT (REA) contributed significantly to the variances of HINT (COMPOSITE), albeit minimally (8.2%). The lack of significant influence of GIN on HINT outcomes fits well with those of the previous studies^10^, ^44^, ^45^ which also found that gap detection threshold did not influence speech recognition performance in quiet and in steady state noise. The lack of pronounced temporal fluctuations in level or spectrum of steady state as used in HINT partly explained this finding.

There have been limited studies which examined the relationship between dichotic listening and speech recognition performance. A study which examined the relationship between the two measures found a strong negative correlation between dichotic score and speech recognition in noise.^46^ It is important to note, however, that the relationship was measured using bivariate correlation, without controlling for other possible confounding factors such as hearing thresholds and cognitive function. In the present study, REA of dichotic listening test was found to contribute to about 9.0% of the variance in HINT (COMPOSITE) after controlling for other tested independent variables. This significant association could be because both measures share some common underlying processes such as attention.

### Contributions of cognitive function to HINT outcomes

The cognitive function as indexed by MoCA uniquely contributed to 5.7% and 18.5% of the variances of the HINT (Q) and HINT (Composite), respectively. The greater contribution of MoCA on HINT (Composite) than HINT (Q) is expected because listening to speech in quiet relies more on speech audibility and is less cognitively taxing. During speech recognition process, speech information is continuously compared with the information stored in long-term memory. If these information match, speech processing and understanding on higher level will occur implicitly and effortlessly.^47^ However, whenever the auditory input is degraded either due to hearing loss or due to noise, additional cognitive-control processes are necessary to support speech recognition.^48^

### Limitations of the study

This study has a few limitations. First, the use of MoCA rather than more specific neuropsychological test battery make it impossible to ascertain which cognitive domains contribute to the speech recognition performance. MoCA as a single measure of cognitive function has, however, been use in previous studies which examined the influence of cognitive functions on speech understanding by older adults.^11^, ^23^ One of the studies which examined the contributions of auditory, cognitive and linguistic abilities on the listening in spatialized noise-sentences (LiSN-S) used MoCA as a measure of cognition. Their results indicated that MoCA predicted three of the LiSN-S outcomes.^11^ Likewise, the use of MyHINT which uses steady-state speech noise did not replicate the most common noise encountered in everyday communication which is largely modulated in nature. Despite that, it is noteworthy to highlight that the fact that MoCA contributed significantly to MyHINT performances suggests that the test is challenging enough to tax the cognitive function during speech recognition tasks in older adults.

## Conclusions

This study examined the contributions of hearing level, measures of central auditory processing, and cognitive status on speech recognition performance in quiet and in noise. Our findings revealed that BE 4FA, BE HFA and cognition were the significant predictors and accounted for about 50% of the variance of speech recognition in quiet. In contrast, HFA, cognitive status as measured by MoCA and REA significantly predicted about 30% of the variance of speech recognition in noise condition. While high frequency hearing was the primary predictor for speech recognition in quiet, MoCA score contributed more than high frequency hearing to the variance of speech recognition in noise. These findings suggest that rehabilitation that geared towards improving cognitive function could help in alleviating some of speech recognition difficulties experienced by older adults, especially, in noisy environment. Additionally, the fact that the included predictors explain only about 20–40% of HINT performance, suggests that there are other relevant predictors that have not been covered in the present study.

## Funding

This study was funded by the Malaysian Ministry of Education research grant (LRGS/BU/2012/UKM-UKM/K/02).

## Conflicts of interest

The authors declare no conflicts of interest.

## References

[1] Dubno J.R., Dirks D.D., Morgan D.E. (1984). Effects of age and mild hearing loss on speech recognition in noise. J Acoust Soc Am.

[2] Working Group on Speech Understanding (1988). Speech understanding and aging. J Acoust Soc Am.

[3] Pichora-Fuller M.K. (2003). Cognitive aging and auditory information processing. Int J Audiol.

[4] Pichora-Fuller M.K., Singh G. (2006). Effects of age on auditory and cognitive processing: implications for hearing aid fitting and audiologic rehabilitation. Trends Amplif.

[5] Syka J. (2002). Plastic changes in the central auditory system after hearing loss, restoration of function, and during learning. Physiol Rev.

[6] Harada C.N., Natelson Love M.C., Triebel K.L. (2013). Normal cognitive aging. Clin Geriatr Med.

[7] Divenyi P.L., Haupt K.M. (1997). Audiological correlates of speech understanding deficits in elderly listeners with mild-to-moderate hearing loss. I. Age and lateral asymmetry effects. Ear Hear.

[8] Goŕdon-Salant S., Fitzgibbons P.J. (1993). Temporal factors and speech recognition performance in young and elderly listeners. J Speech Hear Res.

[9] Gosselin P.A., Gagné J.P. (2011). Older adults expend more listening effort than young adults recognizing audiovisual speech in noise. Int J Audiol.

[10] Schoof T., Rosen S. (2014). The role of auditory and cognitive factors in understanding speech in noise by normal-hearing older listeners. Front Aging Neurosci.

[11] Besser J., Festen J.M., Goverts S.T., Kramer S.E., Pichora-Fuller M.K. (2015). Speech-in-speech listening on the LISN-S test by older adults with good audiograms depends on cognition and hearing acuity at high frequencies. Ear Hear.

[12] Tyler R.S., Summerfield Q., Wood E.J., Fernandes M.A. (1982). Psychoacoustic and phonetic temporal processing in normal and hearing-impaired listeners. J Acoust Soc Am.

[13] Andersson M., Reinvang I., Wehling E., Hugdahl K., Lundervold A.J. (2008). A dichotic listening study of attention control in older adults. Scand J Psychol.

[14] Commodari E., Guarnera M. (2008). Attention and aging. Aging Clin Exp Res.

[15] Astheimer L.B., Sanders L.D. (2009). Listeners modulate temporally selective attention during natural speech processing. Biol Psychol.

[16] Jerger J., Chmiel R., Allen J., Wilson A. (1994). Effects of age and gender on dichotic sentence identification. Ear Hear.

[17] Carter A.S., Noe C.M., Wilson R.H. (2001). Listeners who prefer monaural to binaural hearing aids. J Am Acad Audiol.

[18] Meister H. (2017). Speech audiometry, speech perception, and cognitive functions. HNO.

[19] Akeroyd M.A. (2008). Are individual differences in speech reception related to individual differences in cognitive ability? A survey of twenty experimental studies with normal and hearing-impaired adults. Int J Audiol.

[20] Besser J., Koelewijn T., Zekveld A.A., Kramer S.E., Festen J.M. (2013). How linguistic closure and verbal working memory relate to speech recognition in noise – a review. Trends Amplif.

[21] Humes L.E. (2002). Factors underlying the speech-recognition performance of elderly hearing-aid wearers. J Acoust Soc Am.

[22] Dryden A., Harriet A.A., Henshaw H.A.H.A. (2017). The association between cognitive performance and speech-in-noise perception for adult listeners: a systematic literature review and meta-analysis. Trends Hear.

[23] Zhan Y., Fellows A.M., Qi T., Clavier O.H., Soli S.D., Shi X. (2018). Speech in noise perception as a marker of cognitive impairment in HIV infection. Ear Hear.

[24] Moncrieff D.W., Wertz D. (2008). Auditory rehabilitation for interaural asymmetry: preliminary evidence of improved dichotic listening performance following intensive training. Int J Audiol.

[25] WHO (2007). http://www.who.int/pbd/deafness/hearing_impairment_grades/en/.

[26] Wiley T.L., Cruickshanks K.J., Nondahl D.M., Tweed T.S., Klein R., Klein B.E. (1996). Tympanometric measures in older adults. J Am Acad Audiol.

[27] Mukari S.Z., Keith R.W., Tharpe A.M., Johnson C.D. (2006). Development and standardization of single and double dichotic digit tests in the Malay language. Int J Audiol.

[28] Musiek F.E., Shinn J.B., Jirsa R., Bamiou D.E., Baran J.A., Zaida E. (2005). GIN (gaps-in-noise) test performance in subjects with confirmed central auditory nervous system involvement. Ear Hear.

[29] Quar T.K., Siti Z.M., Noor Alaudin A.W., Rogayah A.R., Marniza O., Nashrah M. (2008). The Malay hearing in noise test. Int J Audiol.

[30] Soli S.D., Wong L.L.N. (2008). Assessment of speech intelligibility in noise with the hearing in noise test. Int J Audiol.

[31] Vaillancourt V., Laroche C., Mayer C., Basque C., Nali M., Eriks-Brophy A. (2005). Adaptation of the HINT (hearing in noise test) for adult Canadian Francophone populations. Int J Audiol.

[32] Sahathevan R., Mohd Ali K., Ellery F., Mohamad N.F., Hamdan N., Mohd Ibrahim (2014). A Bahasa Malaysia version of the Montreal Cognitive Assessment: Validation in stroke. Int Psychogeriatrics.

[33] Nasreddine Z.S., Phillips N.A., Bédirian V., Charbonneau S., Whitehead V., Collin I. (2005). The Montreal Cognitive Assessment MoCA: a brief screening tool for mild cognitive impairment. J Am Geriatr Soc.

[34] Humes L.E., Roberts L. (1990). Speech-recognition difficulties of the hearing-impaired elderly: the contributions of audibility. J Speech Hear Res.

[35] ANSI (1997).

[36] Levitt H., Wang M.C., Reynolds M.C., Walberg H.J. (1989).

[37] Moore B.C., Sek A. (1996). Detection of frequency modulation at low modulation rates: evidence for a mechanism based on phase locking. J Acoust Soc Am.

[38] Feng Y., Yin S., Kiefte M., Wang J. (2010). Temporal resolution in regions of normal hearing and speech perception in noise for adults with sloping high-frequency hearing loss. Ear Hear.

[39] Simon H.J., Yund E.W. (1993). Frequency discrimination in listeners with sensorineural hearing loss. Ear Hear.

[40] Schroder A.C., Viemeister N.F., Nelson D.A. (1994). Intensity discrimination in normal-hearing and hearing-impaired listeners. J Acoust Soc Am.

[41] Silberer A.B., Bentler R., Wu Y.H. (2015). The importance of high-frequency audibility with and without visual cues on speech recognition for listeners with normal hearing. Int J Audiol.

[42] French N.R., Steinberg J.C. (1947). Factors governing the intelligibility of speech sounds. J Acoust Soc Am.

[43] Fletcher H., Galt R. (1950). The perception of speech and its relation to telephony. J Acoust Soc Am.

[44] Helfer K.S., Freyman R.L. (2008). Aging and speech-on-speech masking. Ear Hear.

[45] George E.L.J., Zekveld A.A., Kramer S.E., Goverts S.T., Festen J.M., Houtgast T. (2007). Auditory and nonauditory factors affecting speech reception in noise by older listeners. J Acoust Soc Am.

[46] Lavie L., Banai K., Attias J., Karni A. (2014). How difficult is difficult? Speech perception in noise in the elderly hearing impaired. J Basic Clin Physiol Pharmacol.

[47] Baddeley A. (2003). Working memory and language: an overview. J Commun Disord.

[48] Houtgast T., Festen J.M. (2008). On the auditory and cognitive functions that may explain an individual's elevation of the speech reception threshold in noise. Int J Audiol.

